# Mitigation of intrahepatic cholestasis induced by 17α-ethinylestradiol via nanoformulation of *Silybum marianum* L.

**DOI:** 10.1186/s12906-024-04351-2

**Published:** 2024-01-23

**Authors:** Maha B. Salem, Dina Mostafa Mohammed, Olfat A. Hammam, Mohamed Elzallat

**Affiliations:** 1 Pharmacology Department, Theodor Bilharz Research Institute, P.O. box 30, Warrak El-Hadar, Giza, 12411 Imbaba Egypt; 2 Nutrition and Food Sciences Department, National Research Centre, Dokki, Giza, 12622 Egypt; 3 Pathology Department, Theodor Bilharz Research Institute, P.O. box 30, Warrak El-Hadar, Giza, 12411 Imbaba Egypt; 4 Immunology Department, Theodor Bilharz Research Institute, P.O. box 30, Warrak El-Hadar, Giza, 12411 Imbaba Egypt

**Keywords:** *Silybum Marianium* L., Nanoformulation, Intrahepatic cholestasis, Ethinylestradiol, Bile acid efflux, Inflammation

## Abstract

**Background:**

Cholestasis is an important predisposing factor for hepatocyte damage, liver fibrosis, primary biliary cirrhosis, and even liver failure. *Silybum marianum* L. (SM) plant is used in teas or eaten in some countries due to its antioxidant and hepatoprotective properties. Because of its low and poor oral bioavailability, so we improve the therapeutic activity of *Silybum marianum* L. extract (SM) by studying the potential effects of nanoformulation of *Silybum marianium* L. extract (nano-SM) on 17α-ethinylestradiol (EE)-induced intrahepatic cholestasis.

**Methods:**

Thirty female Sprague-Dawley rats were divided into 5 groups (6 rats/group). Group I: Rats were received the treatment vehicle and served as normal group. Group II:Rats were injected daily with EE (10 mg/kg) for five successive days. Group III-V: Rats were injected daily with EE (10 mg/kg) and treated with either Ursodeoxycholic acid (UDCA) (40 mg/kg), SM (100 mg/kg) and nano-SM (100 mg/kg) orally once/day throughout the trialfor five successive days, respectively.

**Results:**

Nano-SM greatly dampened the increase in serum levels of total and direct bilirubin, alanine aminotransaminase, aspartate aminotransaminase, and alkaline phosphatase caused by EE. Furthermore, nano-SM increased the hepatic contents of reduced glutathione (GSH) and catalase (CAT) and also upregulated the relative hepatic gene expressions of Rho-kinase (ROCK-1), myosin light chain kinase (MLCK), and myosin phosphatase target subunit (MYPT1) compared to the EE-induced group. Administration of nano-SM reduced hepatic lipid peroxidation and downregulated the relative hepatic expressions of the nuclear factor-kappa B (NF-ҡB) and interleukin-1β (IL-1β). In addition, nano-SM improved the histopathological changes induced by EE.

**Conclusion:**

Nano-SM possessed a superior effect over SM, which can be considered an effective protective modality against EE-induced cholestatic liver injury through its antioxidant, anti-inflammatory activities, and enhancing bile acid (BA) efflux.

## Background

Cholestasis is a clinically pathogenic condition in which toxic bile acid (BA) accumulates excessively in the liver [1], autoimmune liver diseases [2], liver cell necrosis, liver fibrosis, primary biliary cirrhosis [3], and even liver failure [4]. Patients with chronic liver disease had a 10.26% overall cholestasis incidence rate [5], and the death rate for cholestasis patients is 7.8% [6].

Synthetic estrogen 17-ethinylestradiol (EE) is often employed to create an experimental model of intrahepatic cholestasis to investigate the underlying molecular mechanism of estrogen-induced cholestasis (EIC). When EE activates the estrogen receptor α, BA transporters are suppressed, and BA synthesis enzymes are altered, resulting in cholestatic liver damage [7, 8]. BA transporter malfunction, or oxidative stress, which may cause inflammation, can be the key contributors to the etiology of intrahepatic cholestasis [9, 10].

Currently, limited effective drugs are accessible for cholestasis therapy. Ursodeoxycholic acid (UDCA) is a safe and effective treatment for cholestasis and can effectively treat 50% of primary biliary cholangitis patients; while 40% of patients showed poor responses, and 5–10% of them suffered from intolerance. Nevertheless, some patients suffering from primary biliary cholangitis still show a progressive disease that requires liver transplantation or, in some cases, can lead to death, despite UDCA treatment [11].Therefore, searching for a new drug avenue for treating or protecting against cholestasis is in great demand.

Natural products have recently been considered a crucial aid in the search for innovative therapeutic drugs to treat and prevent cholestatic liver disorders [12, 13]. Milk thistle, or *Silybum marianum* L. (SM), is a member of the Asteraceae family with purple blooms, light green leaves, and thorny, mallow-like stems [14, 15]. The liver cells regenerate more quickly due to its involvement in expelling poisons that bind to the organ [16]. SM is used as a tea or eaten in several countries in the Middle East and North Africa due to its ability to support patients with liver cirrhosis, alcoholic hepatitis, alcoholic fatty liver, liver toxicity, and viral hepatitis [17–19].

In vitro experiments had been proved that *Silybum marianum* L. had the ability to inhibit T-cell proliferation and proinflammatory cytokine secretion in a dose-dependent manner [20, 21]. In animal models, *Silybum marianum* L. had protective effects on rat or mouse liver against hepatotoxicity in acute ethanol intoxication, carbon tetrachloride, cisplatin, thioacetamide, thallium, D-galactosamine and acetaminophen [22–25].Also, *Silybum marianum* L. had anti-inflammatory and antiproliferative effects on cholestasis-induced hepatic injury in rats [26].

Despite the therapeutic benefits of silymarin, its weak biochemical properties (a lack of solubility in water) and limited bioavailability caused unsatisfactory and inconsistent therapeutic results, which leads to more instances of drug-drug interactions when other concurrent medications are given [27, 28] which led to search for innovative method to solve such problems as using nanotechnology. Nanotechnology offers the chance to complement natural remedies by facilitating the identification, manufacture, and implementation of a wide range of therapeutic strategies to improve health and lessen the severity of various diseases [25]. Accordingly, this study aimed to improve the therapeutic activity of SM by studying the potential protective effects of nano-SM on EE-induced intrahepatic cholestasis in Sprague-Dawley rats.

## Materials and methods

### Preparation of the plant extract

SM seeds were collected at the end of February 2021 from NRC Farm, Cairo, Egypt and identified by Dr. Ahmed Ali Muhammed, Department of Horticultural Crops Technology, National Research Centre (NRC). Herbal specimen was kept at NRC herbarium (voucher specimen #6411). Relevant permissions were obtained for the collection of SM seeds in accordance with relevant institutional, national, and international guidelines and legislation.

The ethanolic extract was carried out, as stated by [29], with various modifications. In brief, 200 g of dried SM seeds were combined with 1 L ml of ethanol (70%) and stirred overnight at room temperature. Then, the ultrasonic probe (160 W power, 20 kHz frequency, 50% pulse; Sonics, Vibra, Cell, USA) was utilized for 15 min. The extract was then split apart by centrifugation at 3000 rpm for 15 min, followed by evaporation of the solvent at 50 °C using a rotary evaporator (Büchi R20, Switzerland). Finally, the extract was collected and kept in a volumetric flask at 20 °C until it was needed for the experiment, and the yield % was recorded.

### Chemical analysis of SM

Silymarin content was detected, as stated by [30]. The total carbohydrate content of plant seeds was evaluated using the phosphomolybdic acid technique, as stated by [31]. The total phenolic content of SM was assessed using the spectrophotometric technique of [32, 33]. Tannin contents were evaluated using the Folin-Ciocalteu reagent technique, as stated by [34]. Crude protein content was estimated by multiplying seed nitrogen content by 6.25. At the time of harvest, dry seed mineral content was assessed to determine the total nitrogen (N) using the micro-Kjeldahl technique [35]. Phosphorus (P) and Potassium (K) were determined and estimated as percentages of dry weight as stated by [36]. The contents of Fe, Mn, and Zn were evaluated using atomic absorption spectrophotometer and were calculated in ppm [37]. established a technique for determining the quantity of fatty acids in seeds. Amino acids were extracted and quantified using Amino Acid Analyzer (AAA 400 INGOs Ltd) according to the methods evaluated by [38, 39]. Total flavonoids in crude extract were determined using the [40] method, modified by [41]. Ascorbic acid (Vitamin C) was determined using the spectrophotometric method of [42].

### Antioxidant assay (DPPH radical scavenging assay)

Using the DPPH radical scavenging test, the antioxidant activity of SM was determined as follows: The antioxidant scavenging activity of SM was investigated using the 1,1-diphenyl-2-picrylhydrazyl free radical (DPPH), which was created by mixing 1.5 mL of different dilutions of SM with 1.5 mL of a 0.2 mM methanolic DPPH solution [43]. After 30 min of incubation at 25 °C, recorded as A (sample) was measured using a spectrophotometer at 520 nm. In the absence of SM, a blank experiment was conducted using the same methodology. Absorbance was recorded as A (blank). The % inhibition of both solutions’ free radical-scavenging activity was estimated using the equation below:

% inhibition = 100 (A (blank)–A (sample)) / A (blank).

The IC50, or the concentration of SM necessary to elicit a 50% reduction in initial DPPH concentration, was used to measure antioxidant activity. As a control, ascorbic acid was used. Each value was measured three times for accuracy.

### Preparation of *Silybum marianium* L. Nanoformulation

The nano-SM was prepared using the solvent Emulsification-Diffusion process with novel modifications, as stated by [44]. The Egyptian Patent Office of the Academy of Scientific Research and Technology has a running patent request on this novel method with number 1956/2020, which contains the details of the nano preparation process and the associated measurement data.

### Nanoparticles measurement techniques

#### Transmission electron microscopy (TEM)

The samples were investigated using a transmission electron microscope (TEM) (JEM − 1234) with a 120 KV operating voltage, a magnification power of 600,000 x, a resolving power of 0.3 nm, a CCD camera, and a programmed heating/cooling facility ranging from − 190 ^0^C to 1000 ^0^C. On a copper grid with a carbon coating, samples were kept [45, 46].

#### Zeta sizer nano ZS

Using a Zeta Sizer Nano ZS (Malvern Instruments Inc., Southborough, MA) at 25 °C, the Z-average hydrodynamic diameter of the samples under investigation was evaluated. The size distribution (by number), polydispersity index (PdI), and ζ-potential of nano-SM were investigated.

### Animals

Female adult Sprague-Dawley rats (100-140 g), aged 8 to 11 weeks, were purchased from the Schistosome Biology Supply Center, Theodor Bilharz Research Institute, Giza, Egypt. Rats were housed under 12 h light & dark cycles with free access to water and food at a temperature of 25 ± 2 °C and humidity of 50 ± 15%.

### Drugs and doses

EE (Folone (R); Misr Company for Pharmaceuticals, El-Asher Men Ramadan, Cairo, Egypt, batch number: 19,606,007), at a dose of 10 mg/kg, was administrated subcutaneously once/day for five days [47]. EE is used to establish the intrahepatic cholestasis model in adult female Sprague Dawley rats according to [48–50].

UDCA (reference drug) (Ursofalk (R); MINAPHARM, under license of Dr. FALK PHARMA-Germany, batch number: KDE1373), at a dose of 40 mg/kg, was freshly prepared in 2%-cremophore (Sigma-Aldrich, USA) and administrated once/day for five days [47].

### Diet composition

The balanced diet, salt mixture and vitamin mixture were prepared according to [51] as described below (Table 1):

**Table 1:** Ingredient composition of the experimental diet

Ingredients	Balanced diet*
**Casein**	120
**Safflower oil**	100
**Sucrose**	230
**Starch**	450.5
**Mix of vitamin**	10
**Mix of Mineral**	30.5
**Cellulose**	50

*gm/kg diet

### Experimental animal design

A total number of 30 female Sprague-Dawley rats were divided into 5 groups (6 rats / group) as described below:

(I) Normal group.
(II) EE-induced intrahepatic cholestasis group.
(III) EE + UDCA-treated group.
(IV) EE + SM-treated group (SM was administered at a dose of 100 mg/kg) [44].
(V) EE + nano-SM-treated group (nano-SM was administered at a dose of 100 mg/kg) [44].

Twenty-four hours after the administration of treatments, rats will be sacrificed by decapitation under sodium pentobarbital (50 mg/kg, i.p.) anesthesia. Sera were collected for liver function assays. The livers were immediately removed for oxidative stress markers assessment and RNA extraction. Part of the livers was fixed in 10% formalin for histopathological and immunohistological studies.

### Liver injury assessment

Using commercial kits (Biodiagnostics, Egypt), serum alanine aminotransferases (ALT), aspartate aminotransferases (AST), alkaline phosphatase (ALP), and total and direct bilirubin were tested.

### Oxidative stress markers assessment

For oxidative stress markers assessment, liver tissue was homogenized, followed by centrifugation for 10 min at 600 g and then for 20 min at 10,000 g at 4 ^o^C. The supernatants were tested for reduced glutathione (GSH), catalase (CAT), and lipid peroxidation, which was expressed by malondialdehyde (MDA) formation, using commercial kits (Biodiagnostics, Egypt).

### Histopathological examination

10% formalin-fixed liver samples were processed and stained with hematoxylin-eosin (H&E) stain. The histopathological changes were examined under a light microscope (magnification x400).

### Real time PCR for BA efflux and inflammatory markers

RNA was extracted from homogenized liver tissues and reverse transcripted to cDNA using QIAamp-RNA-Mini-Kit (Qiagen, Germany) and QuantiTect-Reverse-Transcription-kit (Qiagen, Germany), respectively. Finally, the relative expressions of ROCK, MLCK, MYPT1, NF-ҡB, and IL-1β genes were done using QuantiTect-SYBR-Green-PCR-kit (Qiagen, USA). β-actin was used as a reference gene. Primers were designed by Thermo (Table 2). The expressions of ROCK, MLCK, and MYPT1 are used to assess the ROCK/MLCK/myosin pathway, which is responsible for bile canaliculi (BC) spontaneous contractions and dilations, which are essential for bile acid (BA) efflux [52]. While the expressions of NF-ҡB, and IL-1β are used to assess the inflammatory process, which is one of the key contributors to the etiology of intrahepatic cholestasis [9, 10].

**Table 2:** The primer sequences for real-time PCR

Target gene (s)	Primer sequence
**Rock1**	Forward (F) primer:5’-AGAAAGAGGACTTGATTTCCCCGTGC-3’
	Reverse (R) primer: 5’- ACGGACAAAGCCAGATGGTGGG-3’
**MLCK**	Forward (F) primer:5’-AGAAGTCAAGGAGGTAAAGAATGATGT-3’
	Reverse (R) primer: 5’- CGGGTCGCTTTTCATTGC-3
**MYPT1**	Forward (F) primer:5’-AAGCGCTCCGTCGTCGTCCT-3’
	Reverse (R) primer: 5’- TCCCCGGGAGTAGGCAGAGGT-3’
**NF-κB**	Forward (F) primer: 5’-CTGGTGGACACATACAGGAAGAC-3’
	Reverse (R) primer: 5’-ATAGGCACTGTCTTCTTTCACCTC-3’
**IL-1β**	Forward (F) primer: 5’-GCTGCTACTCATTCACTGGCAA-3’
	Reverse (R) primer: 5’-TGCTGCTGGTGATTCTCTTGTA-3’
**Beta actin**	Forward (F) primer:5’-ACCGTGAAAAGATGACCCAG-3’
	Reverse (R) primer: 5’- TCTCAGCTGTGGTGGTGAAG-3’

### Immunohistochemical evaluations of IL-6

Immunohistochemical evaluations of IL-6 were performed using an anti-IL-6 antibody (Santa Cruz Biotechnology, USA) and DAKO-EnVision-FLEX detection kit [53]. The percentages of positive cytoplasmic staining for IL-6 in 10 fields (magnification x400) were calculated for each rat.

### Statistical analysis

SPSS, software package version 16.0 (Chicago, USA), One-way-ANOVA-test followed by Tukey-*hoc*-test was used to determine the significant difference between the different groups. Results were expressed as mean ± SE and considered significant when the *P* < 0.05.

## Results and discussion

The results of this experiment revealed that nano-SM could improve the therapeutic activity of SM and can be used as an effective modality in protecting against EE-induced-cholestatic liver injury, as the limitations of the SM were resolved by using thisnano-formulation.

### Chemical analysis of SM

**Table 3:** Yield and chemical composition of *Silybum marianum* L. seeds

SM yield of the ethanolic extract			
Plant species	Parts used	Yield/200 g dry plant	
*Silybum marianum* L.	Aerial parts	50	
**Chemical composition of** ***Silybum marianum*** **L. seeds**			
**Constituents**			***Silybum marianum*** **L.**
Silymarin **mg/g**			51.43
Total carbohydrates **%**			43.79
Total phenolic **mg/g**			31.51
Tannin **mg/g**			3.41
Crud protein **%**			24.36
Ascorbic acid (Vitamin C) **mg/g**			2.67
Total Flavonoids **mg/g**			19.95

SM had an extraction yield of **50** g/**200** g dried plant (Table 3), which was corroborated by [54, 55]. Silymarin concentration in SM was **51.43** mg/g. This finding has been supported by other researchers [56–58]. The total carbohydrates were **43.79**%, and the crudeprotein was **24.36**%, with total phenolic, tannin, ascorbic acid, and total flavonoids averaging **31.51** mg/g, **3.41** mg/g, **2.67** mg/g, and **19.95** mg/g, respectively. SM has a protein content of **25–30**% [59–62]. Protein and total carbohydrates are abundant in the seeds. Antioxidant qualities are attributed to flavonoids and phenolic acids due to the presence of hydroxyl groups in their structures; these compounds play a crucial role in the body’s defense mechanism versus oxidative damage produced by endogenous free radicals [63]. Vitamin C (ascorbic acid) is engaged in a variety of metabolic processes and is required for collagen formation; it also improves blood circulation and acts as an antioxidant [64] (Table 3).

**Table 4:** Minerals content, Fatty acids composition (%), and Amino acids composition of *Silybum marianum* (L.) seeds

Minerals content of SM seeds	
Minerals content	Silybum marianum L.
K **%**	5.2
N **%**	3.94
*P* %	0.89
Fe **ppm**	0.91
Zn **ppm**	0.089
Mn **ppm**	0.088
**Fatty acids composition (%) of SM seeds**	
**Fatty acids**	***Silybum marianum*** **L.**
Linoleic acid	48.2
Oleic acid	29.4
Palmitic acid	12.9
Stearic acid	11.8
**Amino acids composition (g-1/kg) of SM seeds**	
**Amino acids**	***Silybum marianum*** **L.**
Phenylalanine	5.79
Tryptophan	0.99
Lysine	4.98
Valine	3.97
Histidine	1.89

Micronutrients, also known as trace elements, are a class of nutrients found in small amounts in the diet. Because of growing evidence of marginal or insufficient intakes among the population, the nutrition community is particularly interested in the trace element composition of diets [65]. According to Table 4, K, N, and P made up **5.2**%, **3.94**%, and **0.89**% of the total SM seed, respectively. Fe, Zn, and Mn concentrations were found to be **0.91**, **0.089**, and **0.088** ppm, respectively. The results agreed with [66, 67], who found that SM seeds contain high mineral content of K, N, P, Fe, Zn, and Mn elements, which are necessary for proper human nutrition.

In Table 4, the primary oils found in SM were linoleic acid, oleic acid, palmitic acid, and stearic acid, according to GC analysis. Linoleic acid, oleic acid, palmitic acid, and stearic acid made up **48.2%**, **29.40%**, **12.90%**, and **11.80**% of the total SM seed, respectively. Seed oil is the plant’s second most significant product, and its fatty acid content is comparable to that of seed oil (linoleic acid > oleic acid > palmitic acid > stearic acid) [68].

The nutritional value of food, particularly protein, is determined not just by its amino acid profile but also by the amounts of essential amino acids present [69]. The amino acid content of SM is shown in Table 4, and it is divided into five categories. SM is a rich source of phenylalanine (**5.79** g^-1^/kg). On the other hand, it is a poor source of tryptophan (**0.99** g^-1^/kg). SM was also shown to have significant levels of lysine, valine, and histidine with **4.98**, **3.97**, and **1.89** g^-1^/kg, respectively. The previous investigation concurred with these findings [67].

### Antioxidassayssay (DPPH radical scavenging assay)

**Fig. 1:**
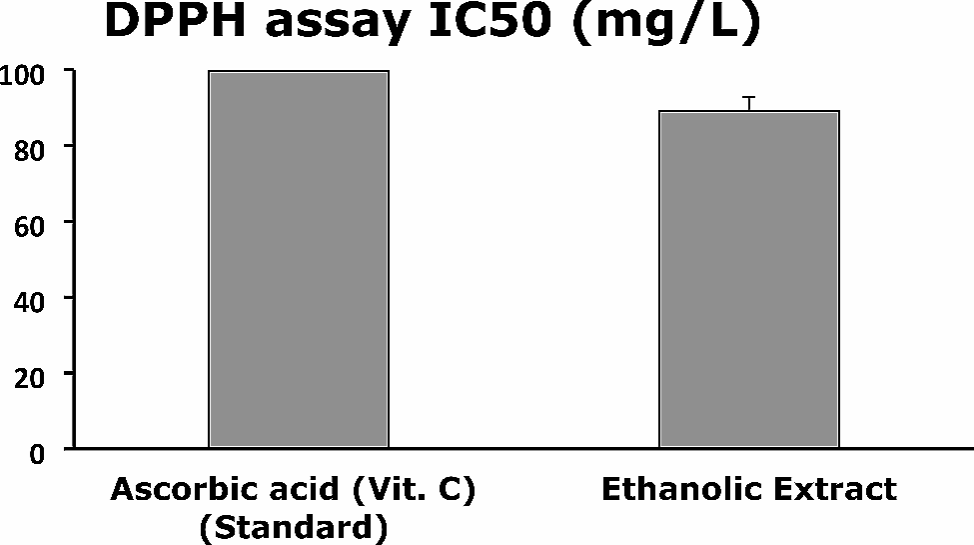
Antioxidant activity of *Silybum marianum* L. using DPPH

The antioxidant activity of SM in Fig. 1 was investigated using the DPPH assay, which showed that the ascorbic acid was **99.7** ± **0.32** mg/L and the ethanolic extract had lower IC_50_ than ascorbic acid, which was **89.1** ± **0.45** mg/L. Due to their potent DPPH radical inhibition, the DPPH radical scavenging capability of SM was shown to have fairly excellent free radical scavenging capacities [70].

### Nanoparticles measurement techniques

#### Transmission Electron Microscopy (TEM) analysis

**Fig. 2:**
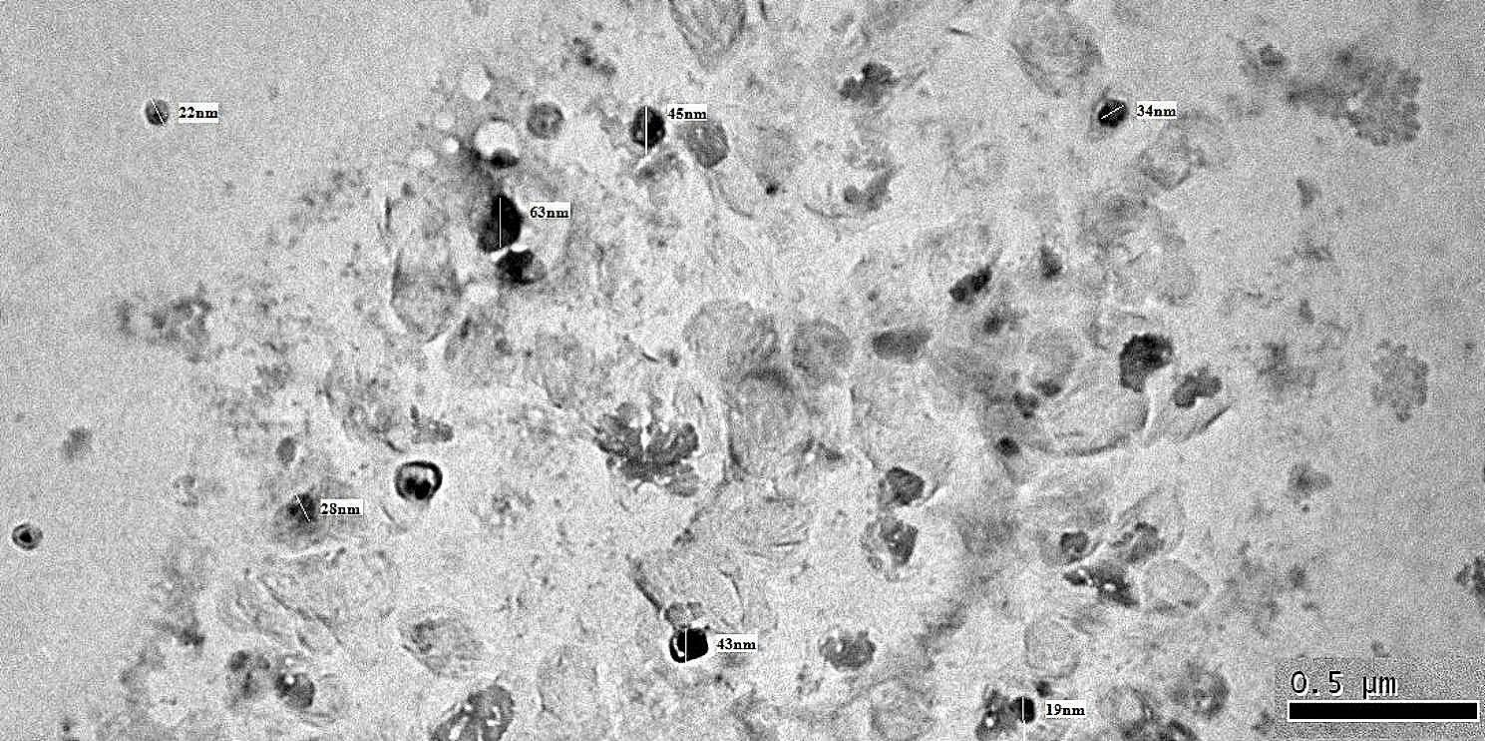
TEM micrographs of *Silybum marianum* L. nanoparticles

Transmission electron microscopy (TEM) has been used as a based technique for investigating the morphology of SM, which revealed spherical vesicles with a diameter of around 50 nm (Fig. 2).

#### Refractindexndex Zeta Sizer

**Fig. 3:**
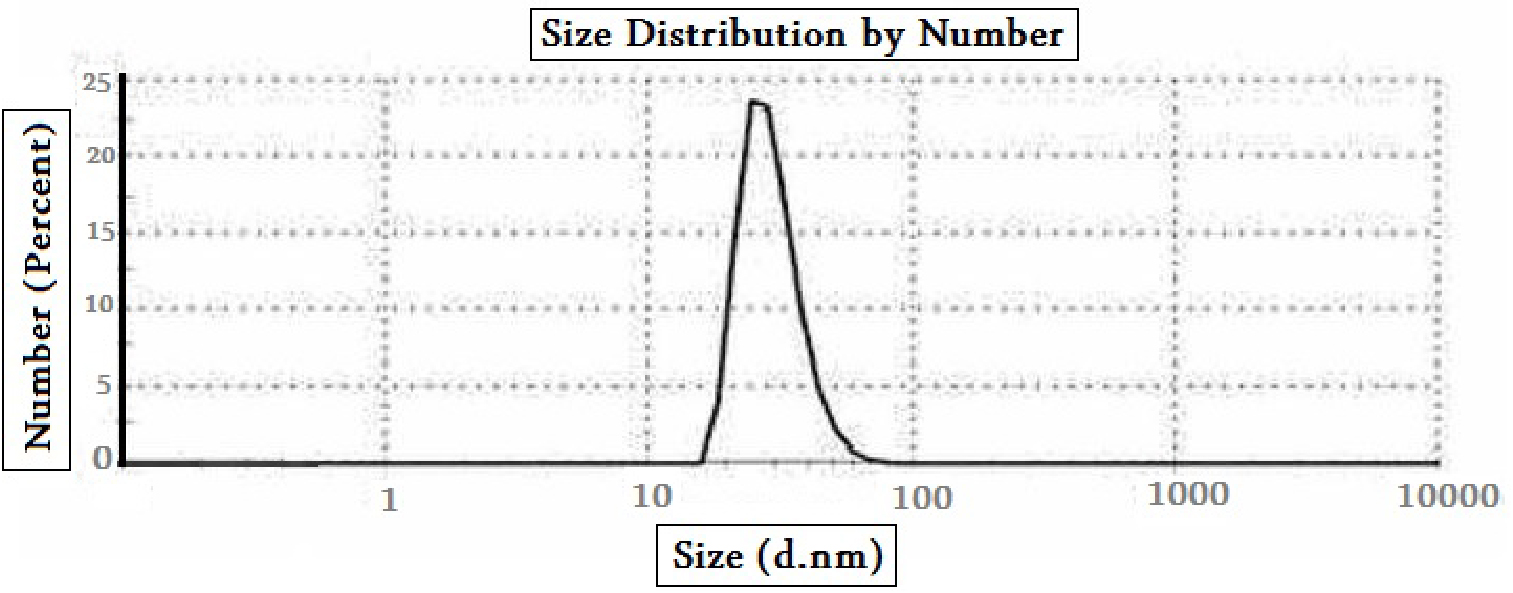
The Zeta Sizer of *Silybum marianum* L.

Nanomaterials differ from their bigger counterparts regarding their physical, chemical, and biological characteristics. Early detection and prevention, better diagnosis, effective treatment, and follow-up of illnesses are all achievable with nanomedicine. The Biopharmaceutics Classification System (BCS) assigns Category IV to SM as a result of its characteristics (such as limited solubility and low permeability) [71, 72]. Therefore, in this study, when converted SM was converted to nanoparticles, the produced nanoparticles with Z-average (d.nm) = 45.27, Pdl = 0.23, and particlesize (d.nm) = 22.67 with % Number = 100% and ζ-potential= -40.40 which exhibited a unique attribute of high surface area to volume ratio according to size distribution, polydispersity index, and zeta-potential data, and their size ranged from 10 to 100 nm, which may significantly affect their interactions with biomolecules and cells (Fig. 3). The size of a colloidal system’s ζ-potential is related to its physical stability; if all particles in a solution have a high ζ-potential (negative or positive), they repel each other, lowering the likelihood of aggregation. Physically constant particles have a ζ-potential larger than 30 mV (positive or negative), but a ζ-potential close to 20 mV (positive or negative) suggests low colloidal suspension stability, and values in the range of 5 mV to + 5 mV indicate fast particle aggregation [73, 74]. Furthermore, these nanoparticles reduce toxicity, retain therapeutic properties, reduce side effects, demonstrate EPR (enhanced permeability and retention) impact, and reduce medication formulation dosage. These factors combine to overcome the limitations of SM, improve its therapeutic activity and make nano-SM an effective modality in protecting against EE-induced-cholestatic liver injury.

### Liver injury Assessment

Cholestasis is a multi-factorial complex disease with various manifestations. Intrahepatic cholestasis is a common symptom of drug-induced liver injury [75]. Estrogen and its metabolites (17β-estradiol) may cause intrahepatic cholestasis in pregnant and pre-menopausal females taking oral contraceptives or hormonal replacement therapy [76]. SM possesses hepatoprotective properties due to its anti-inflammatory, antioxidant, and membrane-stabilizing effects. Moreover, it was also shown to guard against estrogen or taurolithocholate-induced cholestasis through its anti-cholestatic effect, including the bile salt pool size and HCO_3_^-^ output normalization and bile salt transport regulation [77, 78].

**Table 5:** Effect of SM or Nano SM on serum biochemical makers

Animal groups	ALT (IU/mL)	AST (IU/mL)	ALP (IU/mL)	Total Bilirubin (mg/dL)	Direct Bilirubin (mg/dL)
**Normal**	67.23 ± 4.27	133.68 ± 5.78	80.01 ± 1.42	0.51 ± 0.05	0.17 ± 0.03
**EE**	158.07 ± 4.23^a^	240.22 ± 13.75^a^	176.86 ± 6.55^a^	4.07 ± 0.29^a^	1.68 ± 0.22^a^
**EE + UDCA**	95.20 ± 7.88^ab^	159.58 ± 15.00^b^	85.65 ± 4.00^b^	1.13 ± 0.07^b^	0.39 ± 0.07^b^
**EE + SM**	102.99 ± 6.8^ab^	197.99 ± 17.10^ab^	124.04 ± 6.05^abc^	1.98 ± 0.17^abc^	1.22 ± 0.23^abc^
**EE + Nano SM**	82.30 ± 2.95^b^	150.07 ± 4.29^b^	81.74 ± 5.19^bcd^	1.10 ± 0.12^bcd^	0.36 ± 0.07^bcd^

The results are presented as means ± SE. ^a, b, c. d^ Significant difference from normal, EE, EE + UDCA, and EE + SM groups at *p* < 0.05, respectively. ALT: alanine aminotransferase; AST: aspartate aminotransferase; ALP: alkaline phosphatase; EE: 17α-ethinylestradiol; UDCA: ursodeoxycholic acid; SM: *Silybum marianum* L

Table 5 illustrated that the EE-induction of intrahepatic cholestasis led to a noticeable significantly increase (*p* < 0.05)in serum ALT, AST, ALP, and total and direct bilirubin levels by nearly **2.35**, **1.80**, **2.21**, **7.98**, **9.88**, and **7.03**-folds respectively compared to the normal group. This is in agreement with previous studies [79, 80], indicating loss of hepatic cell membrane integrity and leakage of bilirubin and liver enzymes into the blood circulation. Notably, treatment with UDCA significantly suppressed (*p* < 0.05) the EE-induced elevation in serum ALT level by **39.77**% compared to the EE-induced intrahepatic cholestasis group. On the other hand, treatment with UDCA normalized serum AST, ALP, and total and direct bilirubin levels. Administration of SM significantly reduced the elevation (*p* < 0.05) in serum ALT by **34.85**%, AST by **22.78**%, ALP by **29.86**%, total bilirubin by **51.35**%, and direct bilirubin by **27.38**%, compared with the EE-induced intrahepatic cholestasis group. However, treatment with nano-SM resulted in the normalization of liver function markers and bilirubin contents. The reduction of liver enzymes and bilirubin by nano-SM may be due to its membrane-stabilizing activity [77, 78].

### Oxidative stress markers assessment

Oxidative stress and inflammation are crucial in the early induction of liver injury associated with cholestasis [81], where BA accumulation induces liver injury followed by neutrophil recruitment and infiltration [82].

**Fig. 4:**
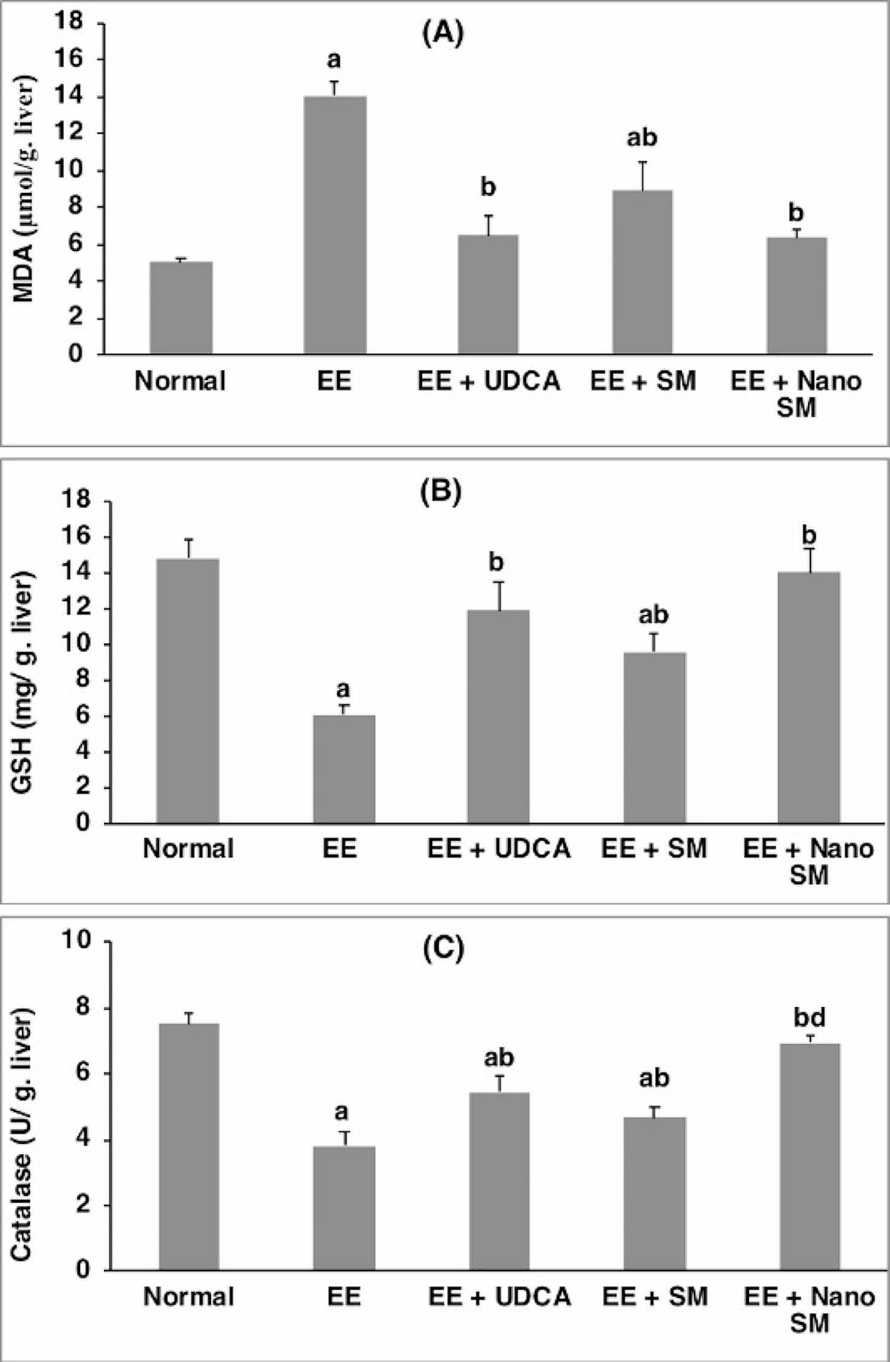
Effect of SM or nano-SM on hepatic (**A**) MDA, (**B**) GSH, and (**C**) CAT contents. The results are presented as means ± SE. ^a, b, c, d^ Significant difference from normal, EE, EE + UDCA, and EE + SM groups at *p* < 0.05, respectively. EE: 17α-ethinylestradiol; UDCA: Ursodeoxycholic acid; SM: *Silybum marianum* L.; MDA: malondialdehyde; GSH: reduced glutathione; CAT: catalase

EE-induced cholestasis resulted in a significant depletion in hepatic GSH and CAT by **2.42** and **1.96**-folds, respectively, along with a significant increase (*p* < 0.05) in lipid peroxidation, which is expressed as hepatic MDA by **2.79**-fold. These results were consistent with the earlier studies of [83, 84]. Nevertheless, treatment with UDCA normalized hepatic GSH and MDA and significantly elevated (*p* < 0.05) hepatic CAT by **53.56**%, compared with the EE-induced intrahepatic cholestasis group. Moreover, treatment with SM resulted in a significant increase (*p* < 0.05) in hepatic GSH and CAT by **56.46**% and **21.41**%, respectively, with a significant reduction (*p* < 0.05) in hepatic lipid peroxidation by **36.64**%, compared with the EE-induced intrahepatic cholestasis group. In addition, nano-SM administration normalized all hepatic oxidative stress markers (Fig. 4), indicating the potent antioxidant activity of the SM and nano-SM. This agreed with previous studies [85, 86] which revealed that SM had antioxidant and hepatoprotective properties.

### Real time PCR for BA efflux and inflammatory marker

Biliary secretion is a multi-step process that includes the translocation of BA across the cell membrane, the cytoplasm, and the canalicular membrane to the canalicular lumen [75]. Because bile canaliculi contraction is essential for BA efflux, Drugs that would improve contractile movement may be a promising candidate for intrahepatic cholestasis treatment.

**Fig. 5:**
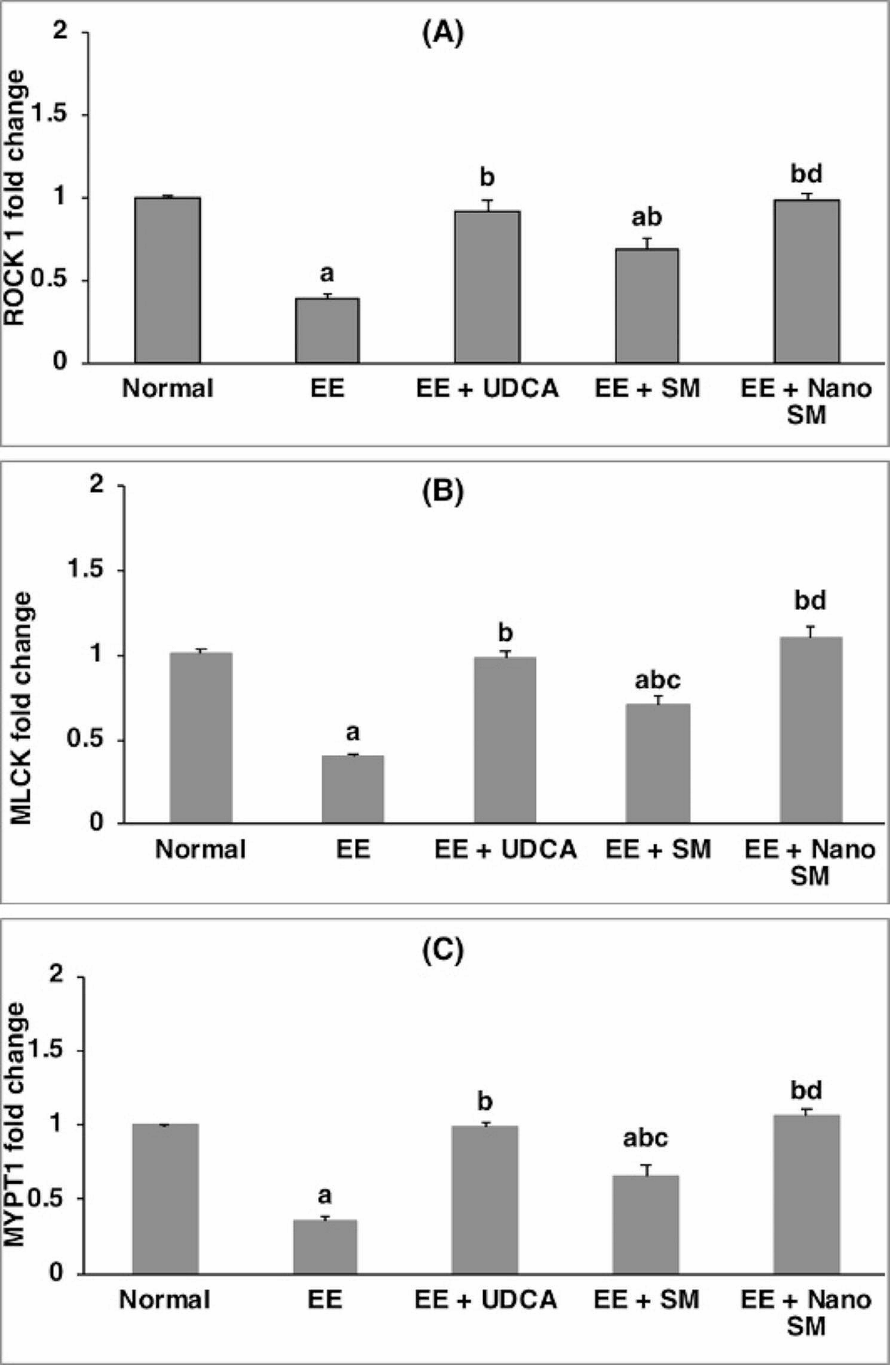
Effect of SM or nano-SM on hepatic (**A**) ROCK 1, (**B**) MLCK, and (**C**) MYPT1 expressions. The results are presented as means ± SE.^a, b, c, d^ Significant difference from normal, EE, EE + UDCA, and EE + SM group sat *p* < 0.05, respectively. EE: 17α-ethinylestradiol; UDCA: Ursodeoxycholic acid; SM: *Silybum marianum* L.; ROCK: Rhokinase 1, MLCK: myosin light chain kinase, MYPT1: myosin phosphatase target subunit

Compared to the normal group, EE deteriorated the BA reflux as manifested bya significant reduction (*p* < 0.05) in ROCK-1, MLCK, and MYPT1 by **2.56**, **2.50**, and **1.39**-folds, respectively (Fig. 5), reflecting bile canaliculidilation and BA accumulation. However, treatment with SM significantly increased (*p* < 0.05) ROCK-1 by **74.36**%, MLCK by **75**% and MYPT1 by **83.33**%, compared with the EE-induced intrahepatic cholestasis group (Fig. 5). On the other hand, administration of UDCA or nano-SM normalized ROCK-1, MLCK, and MYPT1indicating the restoration of normal bile canaliculi contraction leading to normal BA flow. These findings concur with [52], who stated that BA flow depends on bile canaliculi’s spontaneous and rhythmic contractions.

Inflammation occurs in EE-induced intrahepatic cholestasis, and the increased inflammatory cytokines accelerate cholestasis development and progression [87]. NF-κB is a crucial transcription factor that regulates several inflammatory cytokines, including IL-6 and IL-1β [88].

**Fig. 6:**
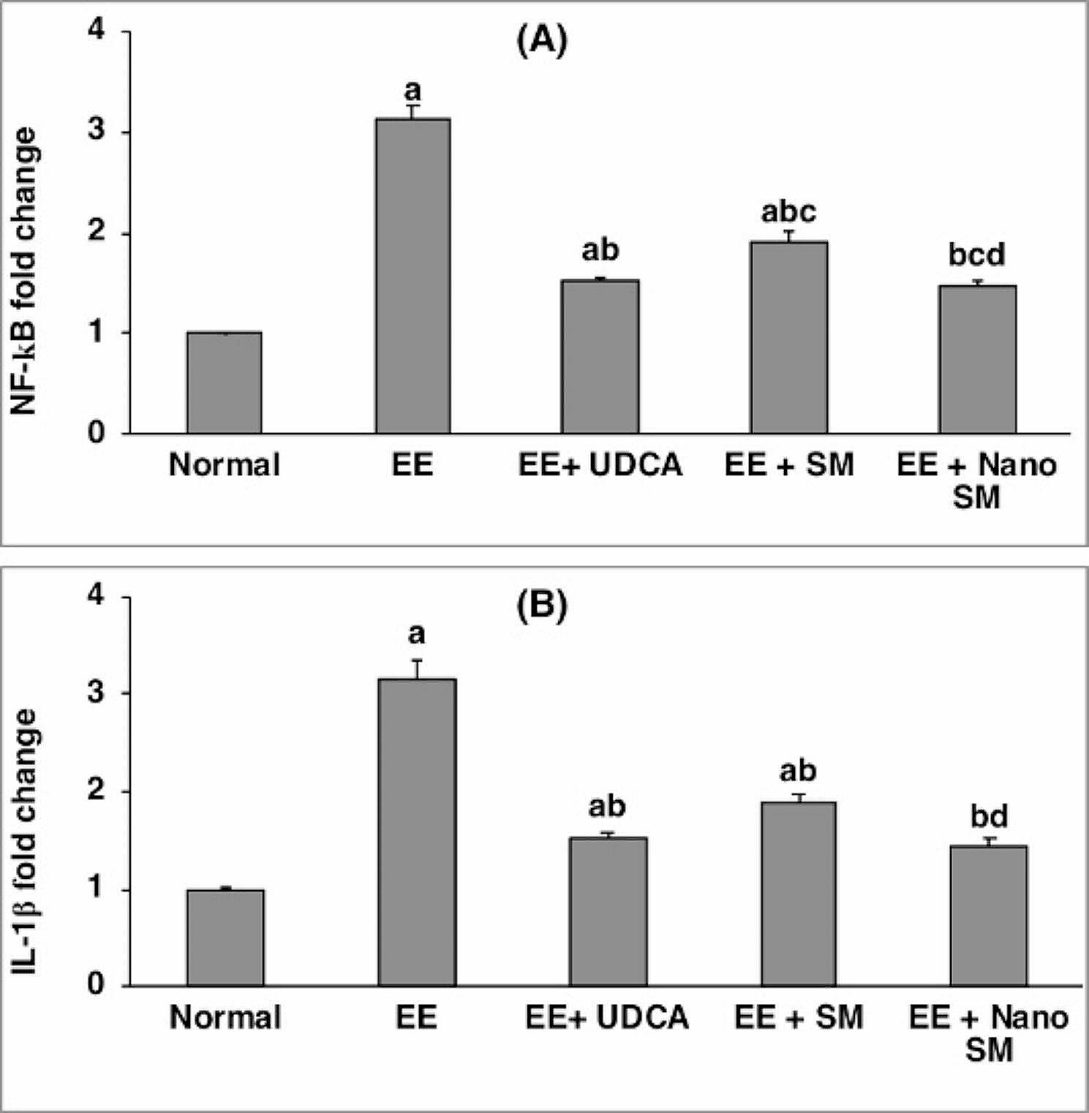
Effect of SM or nano-SM on hepatic (**A**) NF-ҡB and (**B**) IL-1β expressions. The results are presented as means ± SE. ^a, b, c, d^ Significant difference from normal, EE, EE + UDCA, and EE + SM groups at *p* < 0.05, respectively. EE: 17α-ethinylestradiol; UDCA: Ursodeoxycholic acid; SM: *Silybum marianum* L.; NF-kβ: nuclear factor kappa- β; IL-1Ⓡ: Interleukin-1β

EE-induced intrahepatic cholestasis resulted in a significant increase (*p* < 0.05) in hepatic expression of NF-κB and IL-1β by **3.12** and **3.14**-folds, respectively (Fig. 6). Treatment with either UDCA or SM significant decreased (*p* < 0.05) the elevation of NF-κB by **51.28**% and **39.10**%, respectively, and IL-1β by **51.27**% and **39.49**%, respectively, compared with the EE-induced intrahepatic cholestasis group (Fig. 6). Finally, the administration of nano-SM normalized the hepatic expression of both NF-κB and IL-1β (Fig. 6). This agreed with previous studies [89, 90], which revealed that SM has anti-inflammatory properties.

### Histopathological examination

**Fig. 7:**
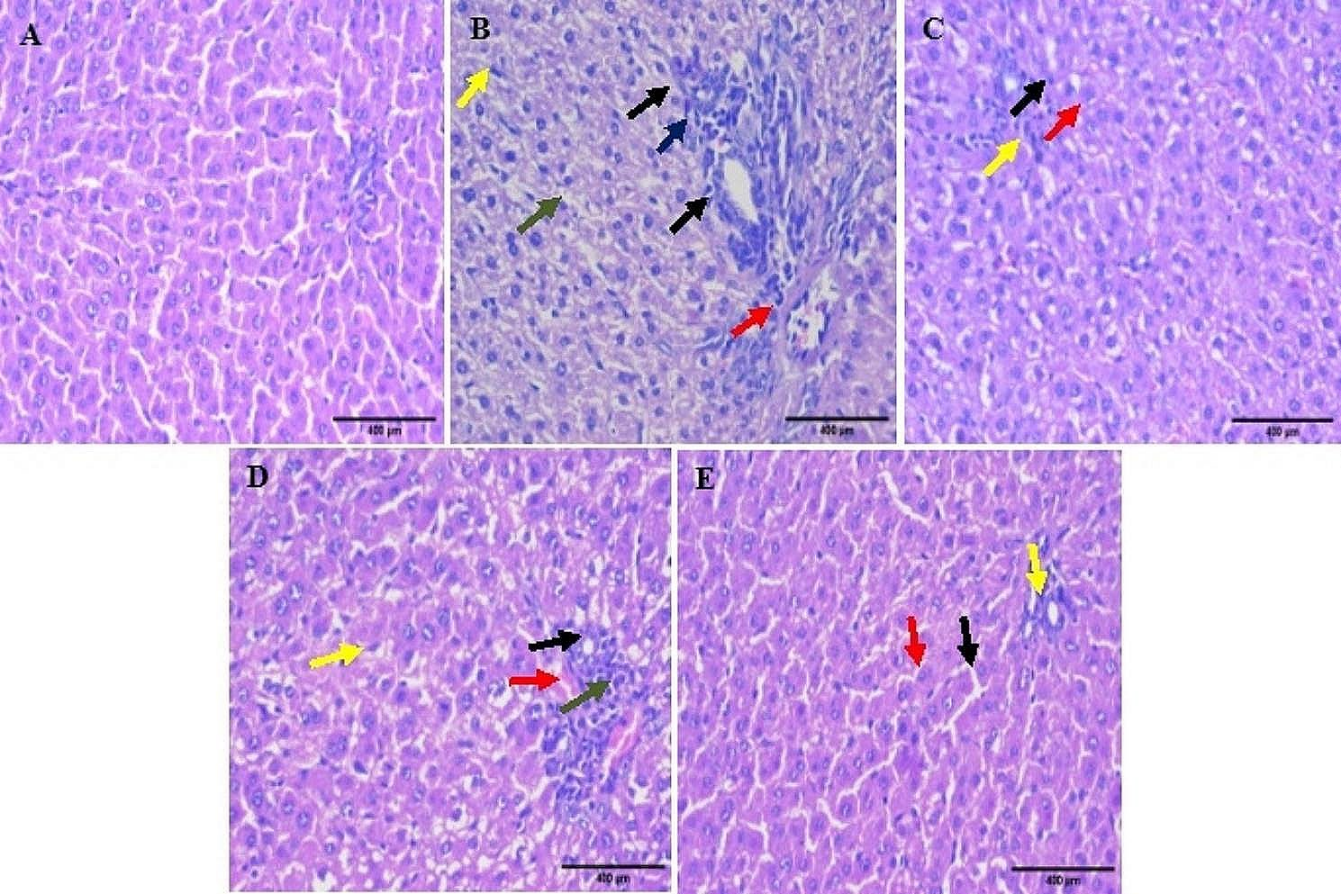
The histopathologic findings of **(A)** normal group showed normal liver lobule including hepatocytes and associated with portal tract, **(B)** EE-induced intrahepatic cholestasis group showed different histopathologic changes including marked hyperemia (red arrow), ductular reaction (proliferation) (black arrow), increase of Kupffer cells (yellow arrow), sinusoidal inflammatory cells infiltrating (green arrow), feathery change (black arrow), portal inflammation (blue arrow), **(C)** UDCA-treated group showed mild hyperaemia (red arrow), bile duct (black arrow), scanty portal inflammation (yellow arrow), **(D)** SM-treated group showed moderate hyperemia (red arrow), ductular reaction (proliferation) (black arrow), increase of Kupffer cells (yellow arrow), moderate portal inflammation (green arrow), **(E)** nano-SM-treated group showed almost normal liver lobule with hepatocytes arranged in thin plate (red arrow), normal sinusoids (red arrow) and associated with portal tract (yellow arrow) (H & E, x400)

The liver section of the normal group revealed normal liver architecture (Fig. 7A). However, EE administration led to severe dilatation and congestion in central and portal veins, severe degeneration in the surrounding hepatocytes, and diffused Kupffer cell proliferation with sinusoidal inflammatory cell infiltrations surrounding the obliterated bile ducts (Fig. 7B). UDCA-treated rats showed mild congestion in the portal vein, mild degeneration in the surrounding hepatocytes, mild Kupffer cell proliferation, and moderate newly formed bile ductules in the portal area (Fig. 7C). Treatment with SM showed moderate congestion in the portal vein, moderate degeneration in the surrounding hepatocytes, moderate Kupffer cell proliferation, and moderate newly formed bile ductules in the portal area (Fig. 7D). Administration of nano-SM revealed almost normal hepatocytes with mild congestion in the central vein and mild inflammatory cell infiltration (Fig. 7E). Our results align with a previous study [91], which demonstrated that SM nanoformulation could improve liver histopathology regarding connective tissue deposition and cellular infiltrates.

### Immunohistochemical evaluations of IL-6

**Fig. 8:**
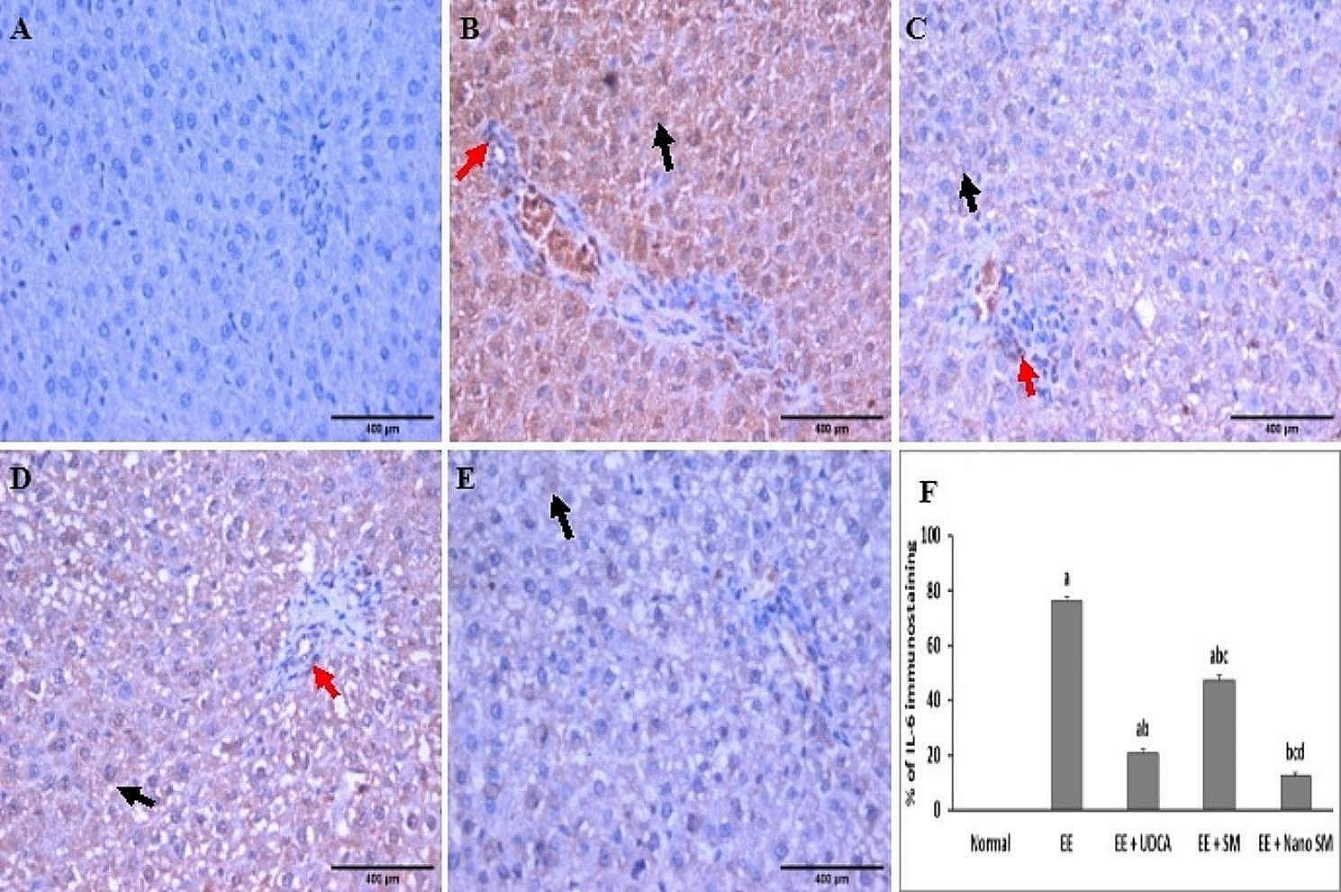
Liver section from **(A)** normal group showed negative expression for IL-6 immunostaining, **(B)** EE-induced intrahepatic cholestasis groupshowed marked positive expression of IL-6 as a brownish cytoplasmic stain in hepatocytes (black arrow), in bile duct lining (red arrow), **(C)** UDCA-treated group showed mild positive expression of IL-6 as a brownish cytoplasmic stain in hepatocytes (black arrow), in bile duct lining (red arrow), **(D)** SM-treated group showed moderate positive expression of IL-6 as a brownish cytoplasmic stain in hepatocytes (black arrow), in bile duct lining (red arrow), **(E)** nano-SM treated group showed mild positive expression of IL-6 as a brownish cytoplasmic stain in hepatocytes (black arrow), **(F)** % of IL-6 immunostaining (DAB, IL 6, x400). The results are presented as means ± SE. ^a, b, c, d^ Significant difference from normal, EE, EE + UDCA, and EE + SM groups at *p* < 0.05, respectively. EE: 17α-ethinylestradiol; UDCA: Ursodeoxycholic acid; SM: *Silybum marianum* L.; IL-6: interleukine-6

The liver section of the normal group showed negative IL-6 expression (Fig. 8A), EE-induced intrahepatic cholestasis group showed a marked increase in the positive IL-6 expression by **76.17**% (Fig. 8B). The UDCA-treated group showed mild positive cytoplasmic expression of IL-6 **(20.83**% & Fig. 8F) in hepatocytes (Fig. 8C). The SM-treated group showed moderate positive cytoplasmic expression of IL-6 (**47.50**% & Fig. 8F) in hepatocytes (Fig. 8D).

Indeed, the administration of nano-SM showed mild positive cytoplasmic expression of IL- (**12.50**% & Fig. 8F) in hepatocytes (Fig. 8E), which confirmed the potent anti-inflammatory activity 0of SM and nano-SM [92].

## Conclusion

The current study has shown that Nanoformulation could improve the therapeutic activity of silymarin and can be considered a promising and effective treatment in protecting against EE-induced cholestatic liver injury, with significant potential for future advancements through its antioxidant and anti-inflammatory activities and enhancing BA efflux. Consequently, Nano-SM may be a potentially beneficial drug for intrahepatic cholestasis prevention. In addition, we also have to take into the consideration the oral bioavailability of Nano-SM in human and the application of drug delivery system for the successful outcomes in clinical trials.

## Data Availability

All data generated and/or analyzed during this study are included in this published article.

## References

[1] Jayappa M, Kumar P, Goyal JP. Prolonged cholestasis after acute viral hepatitis: successfully treated with oral steroid. BMJ Case Reports CP. 2020; 13(5), e234430. 10.1136/bcr-2020-234430 PMC7247385 32444440

[2] Chalifoux SL, Konyn PG, Choi G, Saab S (2017). Extrahepatic manifestations of primary biliary cholangitis. Gut Liver.

[3] Shipovskaya AA, Dudanova OP (2018). Intrahepatic cholestasis in nonalcoholic fatty liver disease. Terapevticheskiiarkhiv.

[4] Jansen PL, Ghallab A, Vartak N, Reif R, Schaap FG, Hampe J, Hengstler JG (2017). The ascending pathophysiology of cholestatic liver disease. Hepatology.

[5] Yang K, Köck K, Sedykh A, Tropsha A, Brouwer KL (2013). An updated review on drug-induced cholestasis: mechanisms and investigation of physicochemical properties and pharmacokinetic parameters. J Pharm Sci.

[6] Björnsson ES. Drug-induced cholestasis. Cholestatic Liver Disease.2014; 13–31.

[7] Yamamoto Y, Moore R, Hess HA, Guo GL, Gonzalez FJ, Korach KS, Negishi M (2006). Estrogen receptor α mediates 17α-ethynylestradiol causing hepatotoxicity. J Biol Chem.

[8] Williamson C, Geenes V (2014). Intrahepatic cholestasis of pregnancy. Obstet Gynecol.

[9] Jin F, Cheng D, Tao JY, Zhang SL, Pang R, Guo YJ, Zhao L (2013). Anti-inflammatory and anti-oxidative effects of corilagin in a rat model of acute cholestasis. BMC Gastroenterol.

[10] Arauz J, Zarco N, Hernández-Aquino E, Galicia-Moreno M, Favari L, Segovia J, Muriel P (2017). Coffee consumption prevents fibrosis in a rat model that mimics secondary biliary cirrhosis in humans. Nutr Res.

[11] Floreani A, Mangini C (2018). Primary biliary cholangitis: Old and novel therapy. Eur J Intern Med.

[12] Ma X, Jiang Y, Zhang W, Wang J, Wang R, Wang L, Zhao Y (2020). Natural products for the prevention and treatment of cholestasis: a review. Phytother Res.

[13] Krepkova LV, Babenko AN, Saybel OL, Lupanova IA, Kuzina OS, Job KM, Enioutina EY. Valuable Hepatoprotective plants-how can we optimize Waste Free uses of such highly versatile resources?. Front Pharmacol. 2021; 12. 10.3389/fphar.2021.738504 PMC8637540 34867345

[14] Ramasamy K, Agarwal R (2008). Multitargeted therapy of cancer by silymarin. Cancer Lett.

[15] Brinda BJ, Zhu HJ, Markowitz JS (2012). A sensitive LC–MS/MS assay for the simultaneous analysis of the major active components of silymarin in human plasma. J Chromatogr B.

[16] Abenavoli L, Izzo AA, Milić N, Cicala C, Santini A, Capasso R (2018). Milk thistle (Silybum marianum): a concise overview on its chemistry, pharmacological, and nutraceutical uses in liver diseases. Phytother Res.

[17] Abu Jadayil S, Tukan SK, Takruri HR (1999). Bioavailability of iron from four different local food plants in Jordan. Plant Foods Hum Nutr.

[18] Ardelean LP, Mihali C, Gavril A, Herman H, Mariasiu T, Popescu C, Hermenean A (2011). Pharmacology of Silybum marianum and its active constituents. Therapeutic activity-part 1. Jurnal Med Aradean.

[19] Koltai T, Fliegel L (2022). Role of silymarin in cancer treatment: facts, hypotheses, and questions. J Evidence-Based Integr Med.

[20] Saller R, Melzer J, Reichling J, Brignoli R, Meier R (2007). An updated systematic review of the pharmacology of silymarin. Forsch Komp Klas Nat.

[21] Ramakrishnan G, Elinos-Baez CM, Jagan S, Augustine TA, Kamaraj S (2009). Silymarin inhibited proliferation and induced apoptosis in hepatic cancer cells. Cell Prolif.

[22] WenYing G, Li J (2012). Chicory seeds: a potential source of nutrition for food and feed. J Anim Plant Sci.

[23] Shaker E, Mahmoud H, Mnaa S (2010). Silymarin, the antioxidant component and Silybum marianum extracts prevent liver damage. Food Chem Toxicol.

[24] Hashem RM, Hassanin KM, Rashed LA, Mahmoud MO, Hassan MG (2016). Effect of silibinin and vitamin E on the ASK1-p38 MAPK pathway in D-galactosamine/lipopolysaccharide induced hepatotoxicity. Experimental Biology and Medicine.

[25] Abou Baker DH, Mohammed DM. Polyphenolic rich fraction of Physalis peruviana calyces and its nano emulsion induce apoptosis by caspase 3 up-regulation and G2/M arrest in hepatocellular carcinoma. Food Bioscience. 2022; 102007.

[26] Alaca N, Özbeyli D, Uslu S, Sahin H, Yigitturk G, Kurtel H, Yegen B. Treatment with milk thistle extract (Silybum marianum), ursodeoxycholic acid, or their combination attenuates cholestatic liver injury in rats: role of the hepatic stem cells. Turkish J Gastroenterol. 2017; 28(6). 10.5152/tjg.2017.16742 29086715

[27] Javed S, Kohli K, Ali M (2011). Reassessing bioavailability of silymarin. Altern Med Rev.

[28] Fenclova M, Novakova A, Viktorova J, Jonatova P, Dzuman Z, Ruml T, Stranska-Zachariasova M (2019). Poor chemical and microbiological quality of the commercial milk thistle-based dietary supplements may account for their reported unsatisfactory and nonreproducible clinical outcomes. Sci Rep.

[29] Shalaby MA, Hamowieh AR (2010). Safety and efficacy of Zingiberofficinale roots on fertility of male diabetic rats. Food Chem Toxicol.

[30] Quaglia MG, Bossu E, Donati E, Mazzanti G, Brandt A (1999). Determination of silymarine in the extract from the dried silybum marianum fruits by high performance liquid chromatography and capillary electrophoresis. J Pharm Biomed Anal.

[31] AOAC CECFP, Association of Official Analytical Chemists. 1999. AOAC International, Arlington, VA.[Newest Panel Design Car Lot 200 W LED Outside Lights for American Parking Lot Lighting]. 2019.

[32] Singleton VL, Rossi JA (1965). Colorimetry of total phenolics with phosphomolybdic-phosphotungstic acid reagents. Am J Enol Viticult.

[33] Jindal KK, Singh RN (1975). Phenolic content in male and female Carica papaya: a possible physiological marker for sex identification of vegetative seedlings. PhysiologiaPlantarum.

[34] Chahardehi AM, Ibrahim D, Sulaiman SF (2009). Antioxidant activity and total phenolic content of some medicinal plants in Urticaceae family. J Appl Biol Sci.

[35] AOAC A (1970). Official methods of analysis.

[36] AOAC International. Association of Official Analytical Chemists. 2005.

[37] Rodríguez-Ruiz J, Belarbi EH, Sanchez JLG, Alonso DL (1998). Rapid simultaneous lipid extraction and transesterification for fatty acid analyses. Biotechnol Tech.

[38] Csomos E, Simon-Sarkadi L (2002). Characterisation of Tokaj wines based on free amino acids and biogenic amines using ion-exchange chromatography. Chromatographia.

[39] Shalabia SE (2011). Bioactive constituents of Atriplexhalimus plant. J Nat Prod.

[40] Zhishen J, Mengcheng T, Jianming W (1999). The determination of flavonoid contents in mulberry and their scavenging effects on superoxide radicals. Food Chem.

[41] Kim DO, Jeong SW, Lee CY (2003). Antioxidant capacity of phenolic phytochemicals from various cultivars of plums. Food Chem.

[42] Jagota SK, Dani HM (1982). A new colorimetric technique for the estimation of vitamin C using Folin phenol reagent. Anal Biochem.

[43] Babili FE, Valentin A, Chatelain C (2013). Lawsoniainermis: its anatomy and its antimalarial, antioxidant and human breast cancer cells MCF7 activities. Pharmaceut Anal Acta.

[44] Ezzat A, Abdelhamid AO, El Awady MK, Abd El Azeem AS, Mohammed DM (2017). The biochemical effects of nano tamoxifen and some bioactive components in experimental breast cancer. Biomed Pharmacother.

[45] Mohammed DM, Elsayed N, Abou Baker DH, Ahmed KA, Sabry BA (2022). Bioactivity and antidiabetic properties of Malva parviflora L. leaves extract and its nano-formulation in streptozotocin-induced diabetic rats. Heliyon.

[46] Mohammed DM, El-Said MM, Badr AN, Abou Baker DH, Hathout AS, Sabry BA. Promising role of Lawsonia inermis L. leaves extract and its nano-formulation as double treatment against aflatoxin toxicity in ulcerated-rats: application in milk beverage. Heliyon, 2023; 9(9). 10.1016/j.heliyon.2023.e19290 PMC10480603 37681189

[47] Ming J, Xu Q, Gao L, Deng Y, Yin J, Zhou Q, Zhang Y (2021). Kinsenoside alleviates 17α-ethinylestradiol-induced cholestatic liver injury in rats by inhibiting inflammatory responses and regulating FXR-mediated bile acid homeostasis. Pharmaceuticals.

[48] Meyers MARK, Slikker WILLIAM, Pascoe GARY, Vore MARY (1980). Characterization of cholestasis induced by estradiol-17-D-glucuronide in the rat. J Pharmacol Exp Ther.

[49] Sano N, Takikawa H, Yamanaka M (1993). Estradiol-17β-glucuronide-induced cholestasis: effects of ursodeoxycholate-3-O-glucuronide and 3, 7-disulfate. J Hepatol.

[50] Muchova L, Vanova K, Suk J, Micuda S, Dolezelova E, Fuksa L, Vitek L (2015). Protective effect of heme oxygenase induction in ethinylestradiol-induced cholestasis. J Cell Mol Med.

[51] Reeves PG, Nielsen FH, Fahey GC Jr. AIN-93 purified diets for laboratory rodents: final report of the American Institute of Nutrition ad hoc writing committee on the reformulation of the AIN-76A rodent diet. 1993. 10.1093/jn/123.11.1939 8229312

[52] Sharanek A, Burban A, Burbank M, Le Guevel R, Li R, Guillouzo A, Guguen-Guillouzo C (2016). Rho-kinase/myosin light chain kinase pathway plays a key role in the impairment of bile canaliculi dynamics induced by cholestatic drugs. Sci Rep.

[53] Hsu SM, Raine L, Fanger H (1981). Use of avidin-biotin-peroxidase complex (ABC) in immunoperoxidase techniques: a comparison between ABC and unlabeled antibody (PAP) procedures. J HistochemCytochem.

[54] Wianowska D, Wiśniewski M (2015). Simplified procedure of silymarin extraction from Silybum marianum L. Gaertner J Chromatographic Sci.

[55] Jahan N, Rehman KU, Basra SMA, Sajid S, Afzal I (2016). Seed enhancement of Silybum marianum and optimization of silymarin extraction. Int J Agri Bio.

[56] Cağdaş E, Kumcuoğlu S, Güventürk S, Tavman Ş. Ultrasound-assisted extraction of silymarin components from milk thistle seeds (Silybum marianum L.). GIDA /. J FOOD. 2011; 36(6).

[57] AbouZid SF, Chen SN, Pauli GF (2016). Silymarin content in Silybum marianum populations growing in Egypt. Ind Crops Prod.

[58] Tayoub G, Sulaiman H, ALorfi M (2018). Quantitative identification of total silymarin in wild Silybum marianum L. by using HPLC. Int J Herb Med.

[59] Wichtl M, Bisset NG. Herbal Drugs andPhytopharmaceuticals, MedpharmSci. Publ. Stuttgart.1994; 225–227.

[60] Wallace SN, Carrier DJ, Clausen EC (2005). Batch solvent extraction of flavanolignans from milk thistle (Silybum marianum L. Gaertner). Phytochemical Analysis: An International Journal of Plant Chemical and Biochemical Techniques.

[61] Abd Raboh FF. Chemical Studies on Milk Thistle Seed as a Novel Source of Human Food ph. D (Doctoral dissertation, Thesis, Fac. of Agric., Tanta Univ., Egypt). 2012.

[62] Azoz SN, Farag HM, Salama AM (2019). Comparative botanical studies two varieties of Silybum marianum (L.) Gaertn.(Asteraceae) in Egypt. Int J Adv Res BiolSci.

[63] Dimitries B (2006). Sources of natural phenolic antioxidant. Trends Food Sci Technol.

[64] George J (2003). Ascorbic acid concentrations in dimethylnitrosamine-induced hepatic fibrosis in rats. ClinicaChimica Acta.

[65] Weaver CM, CM W. PH C, SL R. Effect of milling on trace element and protein content of oats and barley. 1981.

[66] WenWu J, Lin L, Tsai T (2009). Drug-drug interactions of silymarin on the perspective of pharmacokinetics. J Ethnopharmacol.

[67] Apostol L, Iorga CS, Mosoiu C, Mustatea G, Cucu S (2017). Nutrient composition of partially defatted milk thistle seeds. Sci Bull Ser F Biotechnologies.

[68] Bahl JR, Bansal RP, Goel R, Kumar S. Properties of the seed oil of a dwarf cultivar of the pharmaceutical silymarin producing plant Silybum marianum (L.) Gaertn. developed in India. 2015.

[69] Afify AEMM, El-Beltagi HS, Abd El-Salam SM, Omran AA (2012). Protein solubility, digestibility and fractionation after germination of sorghum varieties. PLoS ONE.

[70] Arnao MB (2000). Some methodological problems in the determination of antioxidant activity using chromogen radicals: a practical case. Trends Food Sci Technol.

[71] Di Costanzo A, Angelico R (2019). Formulation strategies for enhancing the bioavailability of silymarin: the state of the art. Molecules.

[72] Pérez-Sánchez A, Cuyàs E, Ruiz-Torres V, Agulló-Chazarra L, Verdura S, González-Álvarez I, Menendez JA (2019). Intestinal permeability study of clinically relevant formulations of silibinin in Caco-2 cell monolayers. Int J Mol Sci.

[73] Mishra A, Ram S, Ghosh G (2009). Dynamic light scattering and optical absorption in biological nanofluids of gold nanoparticles in poly (vinyl pyrrolidone) molecules. J Phys Chem C.

[74] Mohammed DM, Ahmed KA, Desoukey MA, Sabry BA (2022). Assessment of the antiulcer properties of Lawsonia inermis L. leaves and its nano-formulation against prolonged effect of acute ulcer in rats. Toxicol Rep.

[75] Padda MS, Sanchez M, Akhtar AJ, Boyer JL (2011). Drug-induced cholestasis. Hepatology.

[76] Pan X, Jeong H (2015). Estrogen-induced cholestasis leads to repressed CYP2D6 expression in CYP2D6-humanized mice. Mol Pharmacol.

[77] Crocenzi FA, Roma MG (2006). Silymarin as a new hepatoprotective agent in experimental cholestasis: new possibilities for an ancient medication. Curr Med Chem.

[78] Vargas-Mendoza N, Madrigal-Santillán E, Morales-González Á, Esquivel-Soto J, Esquivel-Chirino C, y, González-Rubio MGL, Morales-González JA. Hepatoprotective effect of silymarin. World journal of Hepatology. 2014; 6(3): 144. 10.4254/wjh.v6.i3.144 PMC3959115 24672644

[79] Ozsoy Y, Ozsoy M, Coskun T, Namlı K, Var A, Özyurt B. The effects of L-arginine on liver damage in experimental acute cholestasis an immunohistochemical study. HPB Surgery. 2011; 2011. 10.1155/2011/306069 PMC3132489 21760660

[80] El-Sisi A, Hegazy S, El-Khateeb E. Effects of three different fibrates on intrahepatic cholestasis experimentally induced in rats. PPAR research. 2013; 2013. 10.1155/2013/781348 PMC3753769 23997763

[81] Gonzalez-Sanchez E, Firrincieli D, Housset C, Chignard N (2015). Nuclear receptors in acute and chronic cholestasis. Dig Dis.

[82] Copple BL, Jaeschke H, Klaassen CD. May. Oxidative stress and the pathogenesis of cholestasis. Seminars in liver disease. Volume 30. © Thieme Medical Publishers; 2010. pp. 195–204. 10.1055/s-0030-1253228 20422501

[83] Hussein MA, Abdel-Gawad SM (2010). Protective effect of Jasoniamontana against ethinylestradiol-induced cholestasis in rats. SaudiPharmaceutical J.

[84] Clichici S, David L, Moldovan B, Baldea I, Olteanu D, Filip M, Filip GA (2020). Hepatoprotective effects of silymarin coated gold nanoparticles in experimental cholestasis. Mater Sci Engineering: C.

[85] Taleb A, Ahmad KA, Ihsan AU, Qu J, Lin NA, Hezam K, Qilong D (2018). Antioxidant effects and mechanism of silymarin in oxidative stress induced cardiovascular diseases. Biomed Pharmacother.

[86] Wadie W, Mohamed AH, Masoud MA, Rizk HA, Sayed HM (2021). Protective impact of lycopene on ethinylestradiol-induced cholestasis in rats. Naunyn Schmiedebergs Arch Pharmacol.

[87] Biberoglu E, Kirbas A, Daglar K, Kara O, Karabulut E, Yakut HI, Danisman N (2016). Role of inflammation in intrahepatic cholestasis of pregnancy. J Obstet Gynecol Res.

[88] Giridharan S, Srinivasan M (2018). Mechanisms of NF-κB p65 and strategies for therapeutic manipulation. J Inflamm Res.

[89] Manna SK, Mukhopadhyay A, Van NT, Aggarwal BB (1999). Silymarin suppresses TNF-induced activation of NF-κB, c-Jun N-terminal kinase, and apoptosis. J Immunol.

[90] Zhao X, Wang H, Yang Y, Gou Y, Wang Z, Yang D, Li C (2021). Protective effects of silymarin against D-Gal/LPS-induced organ damage and inflammation in mice. Drug Des Devel Ther.

[91] Younis N, Shaheen MA, Abdallah MH (2016). Silymarin-loaded Eudragit® RS100 nanoparticles improved the ability of silymarin to resolve hepatic fibrosis in bile duct ligated rats. Biomed Pharmacother.

[92] Federico A, Dallio M, Loguercio C (2017). Silymarin/silybin and chronic liver disease: a marriage of many years. Molecules.

